# Windows on the Human Body – *in Vivo* High-Field Magnetic Resonance Research and Applications in Medicine and Psychology

**DOI:** 10.3390/s100605724

**Published:** 2010-06-08

**Authors:** Ewald Moser, Martin Meyerspeer, Florian Ph. S. Fischmeister, Günther Grabner, Herbert Bauer, Siegfried Trattnig

**Affiliations:** 1 MR Center of Excellence, Medical University of Vienna, Lazarettgasse 14, A-1090 Vienna, Austria; E-Mails: martin.meyerspeer@meduniwien.ac.at (M.M.); florian.fischmeister@univie.ac.at (F.Ph.S.F.); guenther.grabner@meduniwien.ac.at (G.G.); siegfried.trattnig@meduniwien.ac.at (S.T.); 2 Center for Medical Physics and Biomedical Engineering, Medical University of Vienna, Währinger Gürtel 18-20, A-1090 Vienna, Austria; 3 Department of Diagnostic Radiology, Medical University of Vienna, Währinger Gürtel 18-20, A-1090 Vienna, Austria; 4 Brain Research Lab, Department of Clinical, Biological and Differential Psychology, Faculty of Psychology, University of Vienna, Liebiggasse 5, A-1010 Vienna, Austria; E-Mail: herbert.bauer@univie.ac.at

**Keywords:** magnetic resonance, ^1^H, ^23^Na, ^31^P, MRI, fMRI, SWI, MRS, EEG, SCP, multi modal imaging, sensors, magnetic field strength, brain, skeletal muscle, joints, cartilage

## Abstract

Analogous to the evolution of biological sensor-systems, the progress in “medical sensor-systems”, *i.e.*, diagnostic procedures, is paradigmatically described. Outstanding highlights of this progress are magnetic resonance imaging (MRI) and spectroscopy (MRS), which enable non-invasive, *in vivo* acquisition of morphological, functional, and metabolic information from the human body with unsurpassed quality. Recent achievements in high and ultra-high field MR (at 3 and 7 Tesla) are described, and representative research applications in Medicine and Psychology in Austria are discussed. Finally, an overview of current and prospective research in multi-modal imaging, potential clinical applications, as well as current limitations and challenges is given.

## 1. Introduction

Over thousands of years, natural sensors (our senses) have been shaped by evolution according to local environments, yielding adjustments and improved coping capabilities for ordinary and new challenges. The performance of human sensor systems is still unsurpassed by technical sensors and sensor systems in many aspects, for example, pattern identification, signal to noise amplification, pattern completion, *etc*. This is likely due to the fact that the development of technical, in our case physical and chemical sensors and sensor systems to enable objective measures, began only about two centuries ago. In the course of medical practice and science a large number of sensing systems, *i.e*., diagnostic techniques, have been developed. Examples are the Riva-Rocci technique, ultrasound imaging, X-ray diagnostics, computed tomography, *etc*. Also in the field of psychology, new research approaches such as psychophysiology and biological psychology have evolved in parallel with classical procedures, *i.e.*, introspection or behavior observation. Since the paradigm shift in the late 1940s and early 1950s from behaviorism to cognitive psychology, biological psychology, especially brain research, became progressively important in all provinces of psychology—the black box of behaviorism is in the process of being opened down to the level of the subcellular processes in the brain.

By employing modern imaging equipment it is now possible to record and store huge data sets of complex information within a short period of time (e.g., several GB in about 15 minutes of functional MRI scanning in a single subject, which will be multiplied by up to a factor of 10 or so during data processing and further multiplied by the number of subjects included in a study). However, in order to make use of the wealth of “objective” information for diagnostic or scientific purpose, all data containing relevant information have to be appropriately reduced and interpreted by a trained clinician or scientist, primarily taking into account the needs of patients. Despite the fact that human brains are sufficiently genetically and anatomically similar to allow them to achieve a variety of tasks along various lines of thought, some brains develop sensor systems and data processing tools and some apply them and interpret the results, leading to a multi-disciplinary world which should help to gain more insight into the complexity of human functions and pathological changes in order to improve our life.

Recent advancements in MR technology [1,2], in particular high-field magnetic resonance imaging and spectroscopy, allow multi-parameter, non-invasive 3D-scanning of the human body [3–5]. This versatile approach enables not only morphological but also functional and metabolic imaging within the same scanner session, as will be described in more detail below. Furthermore, MR technology has been the driving force in medical imaging for over 25 years. This not only stimulates research in MRI and MRS but also in other imaging modalities and even helps to integrate them whenever technically feasible and scientifically meaningful (e.g., MR-EEG, MR-PET).

Using this technology with optimized measurement protocols and advanced data processing enables the function and metabolism of the human senses to be studied and explored, including variability and dysfunction, and how this contributes to human behavior. This, in turn, leads to a better understanding of human cognition and behavior, improves clinical diagnostics, and helps monitoring therapeutic efficacy to improve patients’ quality of life.

Here we have come full circle—by using machinery developed by the human brain to study unconsciously developed abilities, *i.e.*, brain function, by means of complex physico-chemical sensor systems (for example, ultra-high magnetic field MR systems and protocols) and visualization software, whereas medical and psychology experts—familiar with the technology, their potential and shortcomings—have to interpret the data to make them useful for scientific and clinical purposes.

### 

#### Morphological MRI—Techniques and Applications

Morphological MRI of the brain is a powerful method in assessing atrophy in patient cohorts, healthy subjects and normal aging [6,7]. There is no general rule about which contrast should be used to assess morphology, but generally T_1_-weighted images are used to segment neuroanatomical structures such as the hippocampus, gray/white matter boundaries and the ventricles. T_2_ sequences provide high image intensity for structures, which are filled with cerebrospinal fluid (CSF) and is, therefore, suitable to segment the ventricles. MP-RAGE (Magnetization Prepared RApid Gradient Echo) [8] is a very common gradient echo sequence used to acquire T_1_-weighted data. MP-RAGE uses a non-selective 180° pulse to prepare the magnetization in order to achieve a certain contrast followed by a RAGE (RApid Gradient Echo) sequence for image acquisition. The major advantage of MP-RAGE is that it can be used for 3D image acquisition, increasing the signal to noise ratio (SNR) in proportion with the number of slices. Another advantage of MP-RAGE is that gradient echoes are used for data acquisition, which is important in order to keep the Specific Absorption Rate (SAR; see Appendix) low. This is especially important for ultra-high field MR-systems (>3 T). The achievable image resolution using MP-RAGE under clinical conditions, with scan times of about 10-15 min for the brain, is about 0.80 mm isotropic (0.51 mm^3^) on a 3 T system and 0.65 mm isotropic (0.27 mm^3^) on a 7 T system.

In order to acquire T_2_-weighted data, Turbo Spin Echo (TSE) or Fast Spin Echo (FSE) measurement sequences [9] are mainly used, where TSE and FSE are in principal very similar, the difference in naming being a manufacturers’ convention. As T_2_-weighted sequences work with spin echoes, requiring higher RF-energy, the SAR limit can be reached easily on 3 T systems. This problem becomes even worse on 7 T systems where only a small number of slices can be acquired without exceeding the SAR limit. Currently, there are a number of methods under development in order to reduce the RF energy deposition and to acquire T_2_-weighted data on 7 T systems [10].

Susceptibility Weighted Imaging (SWI) is a novel imaging technique that involves both MRI and image processing, exploiting the magnetic susceptibility difference between different tissues to enhance contrast in MR magnitude images [11].

Magnetic susceptibility, which is the degree of magnetization of a substance in response to an external magnetic field B_0_, depends on the tissue’s chemical composition, state, and density. A difference in magnetic susceptibility between neighboring tissues produces a different signal phase that is proportional to the echo time (TE) and the magnetic field strength B_0_. This phase difference can be measured and reconstructed as an image using a SWI protocol. Unlike commonly acquired magnitude images, SWI does not require additional scan time as complex data, *i.e.*, magnitude and phase, are acquired in one scan. Phase images extracted from this complex data are used to enhance contrast in MR magnitude images, but also provide clinically interesting contrast in their own right.

SWI has clear advantages as a high field method, as contrast is proportional to the magnetic field strength B_0_, and a gradient-echo sequence is used for data acquisition which allows high resolution imaging on 7 T systems without running into SAR limits. These features shall enable new insights to be gained into Parkinson’s [12] and Alzheimer’s diseases [13–15], where associated iron accumulations can be mapped with high spatial resolution, and in Multiple Sclerosis, in which lesion-related venous vessels can be visualized. Also, imaging micro bleeds (trauma) or monitoring the neurovascular system of a growing, malignant tumor is feasible. SWI images are sometimes displayed using the minimum intensity projection (mIP) of several slices, which is then called HRBV (high-resolution BOLD venography) [16,17]. To date, SWI has been used in MR venography [16], arterial venous malformations [18], occult venous disease [17], multiple sclerosis [19], tumors [20,21], and brain iron mapping [22–24].

#### Advanced Morphological Imaging of Joints and Cartilage

Recently, high- and ultra-high-field whole-body MR imaging systems have played an increasingly important role in musculoskeletal imaging. However, ultra-high field imaging systems are currently only used in research centers. In clinical settings, 1.5 T scanners have been the gold standard for musculoskeletal MR imaging, but are now increasingly being replaced by 3 T systems. The major advantage of MR imaging systems at 3 T and above is the increased capability to perform high resolution morphological and especially biochemical imaging.

At 1.5 T, most musculoskeletal tissues (e.g., cartilage, muscle, menisci, bone, tendon, ligaments) are rendered with quite a low signal-to-noise ratio (SNR). Modern 3 T systems offer not only an increase in field strength, but also have advanced radiofrequency coil designs, and new imaging sequences. These yield increased SNR, which can be translated into either higher temporal or spatial resolution for improved morphological imaging. The field strength dependent SNR gain is outweighed by the frequency dependency of relaxation times, and there is the need to adjust sequence parameters (e.g., bandwidth, RF pulse parameters) to reduce artifacts, and not exceed SAR limits.

*Imaging the knee:* MRI at 1.5 T already provides very high image quality for knee imaging. Eckstein *et al*., however, evaluated the precision of assessment of cartilage volume, thickness, and surface area of the femorotibial cartilage at 1.5 T and 3 T, and found that results tend to be more reproducible at 3 T than at 1.5 T [25].

Chang *et al*. performed the first *in vivo* 7 T MRI study of the knee. They investigated the trabecular bone microarchitecture, and detected activity-related changes in Olympic fencers compared to healthy controls [26]. It has been shown that dual X-ray measurement of bone density, as currently used for the diagnostics of osteoporosis, is not sufficient to characterize bone quality, because, apart from the bone density, the trabecular bone architecture contributes significantly to the mechanical strength, and thus, fracture risk [27,28]. Such bone structure is routinely characterized by microscopic computed tomography [29,30]. However, *in vivo* trabecular bone imaging is one of the emerging applications for high-resolution morphological MRI; this application—due to the increased sensitivity to susceptibility and the resulting shortened T2* relaxation times—truly benefits from moving to a higher field strength [31,32].

For cartilage imaging, up to a 2.4-fold increase in SNR has been reported in fully balanced steady-state, free-precession imaging comparing 3 T and 7 T. One study detected an increase in SNR from 3 T to 7 T that was significant only for the gradient-echo, but not for the fast spin-echo sequences [33]. In that comparison, however, scan parameters for the fast spin-echo sequence had to be modified due to SAR limits, which resulted in incomplete coverage of the knee joint, extensive artifacts, and a less effective fat saturation. Contrast-to-noise ratio (CNR) and image quality were increased for the gradient-echo and decreased for the fast spin-echo sequences. When comparing 3 T and 7 T, the level of confidence for diagnosing cartilage lesions was higher in the gradient-echo and lower in the fast spin-echo sequences; however, overall, the fast spin-echo sequences at 3 T had the highest confidence score. Evaluation of bone marrow edema was decreased at 7 T due to the limited performance of the fast spin-echo sequences.

Kraff *et al*. have performed a comparison between knee imaging at 1.5 T and 7 T and also reported that, due to the SAR limitations with the fast spin-echo sequences, subsequent measurements were needed for complete coverage of the knee joint [34]. They also confirmed that bone marrow edema was better visualized at 1.5 T than at 7 T. To date, no study has verified a statistically significant increase in diagnostic accuracy for the detection of common pathologies in the knee when moving to MR scanners with field strengths above 3 T.

*Imaging the ankle:* Bauer *et al*. performed trabecular bone imaging of the calcaneus and concluded that 3 T results were more accurate than those calculated from the 1.5 T [35]. Notably, the advances in visualizing trabecular bone structure were partially SNR-independent. They assumed that the better performance at 3 T might have been due to increased sensitivity to susceptibility, enhancing the visualization of thin trabecular structures. The same group published data indicating that ankle abnormalities are better visualised at 3 T than at 1.5 T, and applying parallel imaging reduced the scan time by 56% [36].

Another group confirmed that image quality for imaging the ankle was significantly higher at 3 T than at 1.5 T [37]. In two of four readers, the sensitivity in detecting cartilaginous pathology at the ankle joint was enhanced significantly by imaging at 3 T compared to 1.5 T, using a fast spin-echo sequence. Differences between 1.5 T and 3 T when balanced Steady-State Free Precession (bSSFP) and the Spoiled Gradient Recalled (SPGR) sequences were used were insignificant, based on ratings by all the radiologists in that study. The sensitivities for detecting tendon pathologies were significantly higher at 3 T as opposed to 1.5 T for two of the radiologists in the same study. Specificities were similar for all readers at 1.5 T and 3.0 T.

Imaging the ankle at 7 T is mainly limited by the lack of commercially available, dedicated ankle coils. Thus, one group used an 8-channel head array coil for performing trabecular bone imaging at 7 T, and found that the relative noise enhancement factor was lower at 7 T than at 3 T for parallel imaging acceleration factors higher than three [38,39].

*Imaging the wrist:* The wrist is an especially challenging region because of the small size of the relevant anatomic structures, such as the intrinsic and extrinsic ligaments, the articular cartilage, and the Triangular Fibro Cartilage Complex (TFCC). Sensitivity, specificity, and accuracy for imaging the TFCC pathology are higher at 3 T than 1.5 T [40]. In addition, trabecular bone imaging has already been performed at the wrist using 3 T MR scanners, which resulted in a 16-fold increase in SNR as compared to 1.5 T and a maximum spatial resolution of 0.20 × 0.20 × 2.00 mm^3^ [41].

Farooki *et al*. were the first to image the wrist above 3 T; they qualitatively compared 1.5 T with 8 T and concluded that 8 T was superior for the visualization of the infrastructure of the median nerve and in the definition of the boundaries of the carpal tunnel [42]. Other authors went on to quantify the difference between 1.5 T and 7 T MRI of the wrist and found that SNR was about five times higher in tendon, bone, muscle, and nerve [43]. These authors achieved a maximum spatial resolution of 0.16 × 0.16 × 1.50 mm^3^.

The New York University research group uses an in-house built 8-channel receive and 4-channel transmit RF-coil for wrist imaging, which provides the possibility to perform parallel imaging; the maximum spatial resolution they achieved *in vivo* was 0.08 × 0.08 × 2.00 mm^3^ [44].

In summary, morphological MR imaging at 3 T has already been proven to be superior to 1.5 T in many brain and musculoskeletal imaging applications and is widely used in the clinical routine; nevertheless, imaging at 3 T continues to improve. At 7 T, initial studies have demonstrated the great potential of those systems for ultra-high resolution imaging, but several technical challenges remain to be solved. In the future, we hope to be able to fully realize the benefits of high- and ultra-high-field imaging by combining ultra-high resolution morphological imaging with functional imaging techniques, not only in the brain but also in the small joints of the human body.

#### Functional MRI—Techniques and Applications

During the second half of the last century biology, in particular brain research, became increasingly important in the field of psychology. A new scientific discipline evolved—Cognitive Neuroscience. The rapid growth of knowledge in this discipline has been tellingly documented already by four books entitled “The Cognitive Neurosciences”, edited by Michael Gazzaniga between 1995 and 2009. Two dominant approaches, pursuing ultimately the same aim, are manifest in these works: the first begins with sub-cellular and cellular processes in order to explain functions of small and finally large scale neuronal networks, while the other observes activities of large scale networks during experimentally defined behavioural and cognitive challenges, subdividing them into smaller networks to describe their interconnectivity and interaction. Both utilize the knowledge gained by the other approach.

The latter strategy has been driven by the development of powerful imaging techniques, in particular MRI, fMRI, MRS and PET. These ‘sensor-systems’ have added a new dimension to the data as gathered previously with more simple methods like electrophysiology, providing highly resolved localization of distributed processes. This feature initiated not only the study of whole brain sensory systems but also widely distributed processing systems, that is neuronal networks that mediate complex mental processes such as language, spatial ability, emotion, empathy, *etc*.

The most widely used contrast mechanism in the mapping of brain activation is the so-called BOLD (blood-oxygen level dependent) contrast, which is based on blood oxygenation changes in the venous vessels and capillaries following electric activity of nerve cells and related metabolic changes [45]. In BOLD-based functional MRI, ultra-fast echo-planar imaging (EPI) is the workhorse for functional studies. Time-series of rapid whole- or part-brain imaging slabs are recorded during rest and/or task performance, technically limited by temporal and spatial resolution, SNR and physiological artifacts. Major artifacts result from intra-voxel signal dephasing [46–48], voxel blurring due to broadened point-spread function (PSF), physiological artifacts due to breathing and heart-beat, and gross head motion. The consequences of these artifacts and the correction schemes which may be applied are different in different brain areas as well as being dependent of magnetic field strength and scanner performance, leading to the need for specifically optimized measurement protocols (e.g., Robinson, Moser and Peper, in [5]). Cortical regions, due to better magnetic homogeneity than subcortial or limbic areas, are typically scanned with 27–64 mm^3^ nominal resolution, resulting in a temporal resolution (TR) of about 3 s for a 20-slice slab. In studies of emotion, for example, high-resolution EPI with ≤10 mm^3^ (3 T) or 1 mm^3^ (7 T) voxels should be used, however, this may reduce temporal resolution to >5 s or brain coverage (also depending on the gradient system employed). In studies where high temporal resolution is required (TR ≥ 100 ms), Brain Activity Movies, or BAM’s [49], may be generated. Such rapid acquisition reduces the SNR per unit time, however, as well as the brain coverage that may be achieved. Functional connectivity data may be collected during rest/non-activity using EPI time series [50]. Spontaneous BOLD-signal fluctuations may indicate resting-state networks in the brain [51–53], and also pathological disturbance [54]. Image preprocessing is necessary to reduce artifacts (e.g., Weissenbacher *et al*. [55]) and statistical mapping to either create brain activity maps or visualize white matter bundles. Finally, software to co-register and map functional onto anatomical data may be used to help visualize the wealth of information appropriately.

Research on unconscious visual processes and their capacity to influence human behaviour has a long and controversial history within Psychology (see Kouider *et al*. [56] for a review). An influential view in the Cognitive Neurosciences proposes that such unaware processing of stimuli can occur in sub-cortical structures without any significant involvement of the cortex, while these processes may in turn modulate cortical activity [57]. Other models argue for an essential role of long-distance networks linking, e.g., the higher sensory areas and the prefrontal and parietal cortex [58], or link awareness to early activations within primary sensory areas or functionally specialized brain areas such as the fusiform face area (FFA) [59].

Experimentally, a reduction of conscious awareness can be achieved by reducing the stimulus presentation time below the individual visual sensory threshold. Such an approach enables the investigation of the modulation of brain activity induced by truly sub- (unconscious) and superliminally (conscious) presented neutral stimuli.

#### Multi-Modal Integration at High Magnetic Field: MRI, fMRI and Slow Cortical Potentials (SCP)

Although modern high-field MR scanners offer TRs down to 100 msec, high temporal resolution comes at the cost of reduced spatial resolution and/or brain coverage. This fact initiated efforts to co-record fMRI and the electroencephalogram (EEG) in parallel; the more so because electroencephalography has also evolved into a functional brain imaging technique. Algorithms have been developed and tuned to estimate sources of brain activity based on scalp potential distributions captured via multi-channel EEG recording, however, with the restriction to activities within the cerebral cortex [60,61]. Additionally, EEG is certainly closer linked to the neuronal activity since it is composed of a sum of neuronal field potentials (albeit a complex one), whereas the BOLD signal indirectly mimics neuronal activity via blood oxygen consumption showing an individual but constant delay of 3 s to 8 s in the various brain regions. Parallel recording of fMRI and EEG, therefore, enables short latency imaging with the aid of the fMRI’s high-resolution localization feature. This way both the temporal and spatial course of brain activity can be traced.

Since the first publication of the simultaneous recording of electroencephalogram (EEG) and BOLD contrasted MRI in 1993 [62] great efforts have been made to turn this powerful tool into a standardized technique. However, the technical problems arising from the integration of these two techniques are manifold in terms of developing suitable amplifiers and procedures to correct for MR related artifacts [63]. While MR-compatible EEG amplifiers are becoming increasingly available, elimination of scanner-induced artifacts in the EEG recordings is still challenging. Basically, these artifacts can be divided into two groups: (1) artifacts related to the cardiac pulse, which causes movements of the electrodes within the static magnetic field (B_0_) of the MR-scanner [64] and (2) artefacts produced by the fast switching of the B_0_-gradients [65]. For event-related potentials (ERP), the efficacy of several software algorithms in the compensation of these artifacts has been shown repeatedly (for a review see Ritter & Villringer [66]) allowing the method to become an essential tool in the investigation of neuronal correlates of cognitive functions [63].

Slow cortical potentials (SCP), however, form a special class of ERPs that accompany temporally extended (up to 10–20 s) sensory/cognitive processes. Unlike the regular ERPs, SCPs neither show specific peaks nor a well-structured temporal course but are comprised of spatially and temporally extended waveforms in the frequency range far below 1 Hz—therefore also referred to as direct-current EEG (DC-EEG). While the neural origin of these potential changes is the same as that of ERPs, they are also tightly related to post-synaptic potentials (PSPs), but possibly not only generated by a superposition of many short-living PSPs, but also of long-lasting PSPs. Furthermore, contributions of astroglia-depolarizations possibly induced by neuron-glia communication [67,68] have been shown. Although a complete and coherent view of the neural basis of SCPs is missing, work on animal models has shown a tight correlation between the fMRI BOLD signal and local field potentials measured extracellularly within the cortex (for a review see Logothetis [69]). In humans, this relationship has been shown by Lamm *et al*. [70] and Khader *et al*. [71], using spatial imagery tasks, and by He *et al*. [72] studying spontaneous fluctuations of the BOLD signal.

The studies cited above suggest a tight relationship between SCP-variations and the BOLD response. However, these studies did not record both signals simultaneously. fMRI and EEG data were acquired separately since: (1) MR-compatible EEG amplifiers capable of recording DC-EEG were not available until recently, and (2), because existing scanner-artifact reduction methods would fail for SCPs since they would suppress the DC component of the EEG-signal. In the Results section we present an approach where NeuroConn Inc. (Ilmenau, Germany), in close collaboration with the University of Vienna and the Medical University of Vienna, developed an adequate artifact compensation procedure for simultaneous SCP and fMRI data acquisition.

#### Advanced Functional ^23^Na-Imaging of Articular Cartilage

Articular cartilage consists mainly of an extracellular matrix made of type II collagen, proteoglycan, chondrocytes, and water [73]. Proteoglycan (PG) is made of a linear protein core to which many glycoproteins known as glycosaminoglycans (GAG) [74] are attached. Proteoglycan serves to cross-link the collagen fibrils in the extracellular matrix to provide both compressive and tensile strength to the matrix. In addition, the proteoglycan links restrict fluid flow through the matrix and thereby increase resistance to structural deformation.

The sulfate and carboxyl groups of the glycosaminoglycans impart a negative fixed charge density (FCD) to the matrix. These negative ions attract positive counter-ions (sodium) and water molecules and provide a strong electrostatic repulsive force between the proteoglycans. These osmotic and electrostatic forces are responsible for the swelling pressure of cartilage. The configuration of the PG macromolecules also contributes to the resistance of the matrix to the passage of water molecules and hence affects the mechanics of the cartilage in this fashion.

The onset of osteoarthritis (OA) is understood to be associated primarily with biochemical phenomena. The ability to quantify these molecular changes using novel sensors will provide a tool for the early diagnosis of osteoarthritis and treatment monitoring. The loss of proteoglycan from the extracellular matrix has been hypothesized to be the event that initiates the onset of OA [75,76].

The loss of proteoglycan with the onset of OA results in a reduction of FCD in cartilage. Maroudas *et al*. have shown that the fixed charge density (FCD) of cartilage is correlated to the GAG content of cartilage [77]. Since the FCD is counter-balanced by the Na ions, loss of PG (hence GAG and FCD) due to cartilage degeneration results in the loss of sodium ions from the tissue. The loss of the negatively charged PG lowers the FCD in the tissue, thereby releasing positively charged sodium ions.

Sodium (^23^Na) MR imaging has been validated as a quantitative method of computing FCD and, hence, proteoglycan content, in healthy humans [78–80]. Healthy human cartilage FCD ranges from a concentration of 50 mM to 250 mM, depending on the age and location in the tissue [81]. As demonstrated in controlled cartilage degradation experiments [78,82], the sensitivity of sodium MR imaging is adequate for detecting small changes in proteoglycan content on the order of 5%. Sodium MRI experiments were performed on the knee cartilage of healthy as well as early stage OA patients at 4 T and demonstrated the feasibility of sodium MRI in computing PG loss in early stage OA. [79]

The major advantage of ^23^Na MRI, especially of cartilage, is that it is highly specific to PG content and, since the sodium from surrounding structures in the joint is low (<50 mM), cartilage can be visualized with very high contrast without the requirement for any exogenous contrast agent such as that in dGEMRIC [83]. It can be used to quantify early molecular changes in osteoarthritis.

The disadvantage of sodium MRI is that it requires field strengths of >3 T to obtain quality sodium images that enable accurate quantification of cartilage FCD. Furthermore, due to the limitations of gradient strengths and other hardware requirements, most of the sodium imaging experiments reviewed here employed echo times of >2 ms. Since the fast T_2_ decay of cartilage lies in the range 1–2 ms, substantial signal is lost before the acquisition. This is a major contributor to the low SNR of sodium compared with conventional proton MRI. Additionally, the sodium gyromagnetic ratio (γ) is one-quarter that of protons, meaning that sodium MRI requires four times stronger gradients to obtain images with the same resolution to that of proton MRI. Resolution, however, is not only a function of the gyromagnetic ratio but also of the receiver bandwidth. The MR sensitivity for ^23^Na is only 9.2% of that of protons (^1^H), and the *in vivo* concentration is ∼360 times lower than the *in vivo* water proton concentration. The combination of these factors results in a ^23^Na signal which is approximately 4000 times smaller than the ^1^H signal.

#### *In Vivo* Magnetic Resonance Spectroscopy

Multi-nuclear magnetic resonance spectroscopy (MRS) is a non-invasive tool for investigating metabolism *in vivo*. Its applications range from investigation of normal physiology in healthy humans and studying metabolic changes under the influence of ageing, exercise, nutrition or medication [84–87] to the diagnosis of a wide range of diseases which include, but are not limited to, metabolic and neurological disorders (e.g., diabetes, myopathies, Parkinson’s disease, epilepsy), psychiatric disorders, and many forms of cancer (e.g., in the brain, breast or prostate) [88–90]. *In vivo* magnetic resonance is most commonly associated with its use as an imaging modality for clinical diagnostics (MRI, see previous sections).

MRS is based on the same physical principle as MRI and can be performed on the same equipment; however an additional component of information is obtained from the tissue under investigation: the chemical environment, *i.e*., which molecule or functional group the observed nuclei is surrounded by, can be distinguished via a shift in resonance frequency caused by the molecules’ electron configuration, which leads to shielding of the magnetic field. Additionally, spatial information can be retained to various extents, from no specific localization information, beyond the radio frequency (RF) coil’s position for signal detection, through one dimensional and single voxel localization up to full 3D chemical shift imaging, capable of generating a map of spectra for each point on a three dimensional grid. Of course, the choice of methods is governed by a trade-off between sensitivity, temporal and spatial resolution (both influencing specificity) and total measurement time, limited by motion artifacts, patient compliance and potentially also availability of the scanner. Using ^1^H MRS it is possible to quantify total creatine, trimethylammonium (TMA) compounds, intra- and extramyocellular lipids (IMCL/EMCL) in muscle, involved in the pathogenesis of insulin resistance and type-2 diabetes [91,92]. The metabolites *N*-acetylaspartate (NAA), creatine, choline, inositol, glutamine, glutamate, γ-aminobutyric acid (GABA), lactate and others are accessible when ^1^H MRS is applied to brain [93–97], rendering the method valuable in the study of physiological metabolism and pathologies, as the metabolites’ concentrations, typically abundant in the millimolar range, change [90–100].

MRS is not limited to the hydrogen nucleus. Other physiologically relevant nuclei may be measured by transmitting the RF pulses with a frequency specific to the particular nucleus. This requires dedicated antennas (RF coils) specifically designed to match the resonance frequency of the nucleus and the given magnetic field strength *B*_0_. This offers a wider view on the metabolism and state of tissue under investigation, as either a different set of metabolites can be observed or molecular properties (e.g., binding to cell walls, orientation effects) are exploited to obtain specific information. Multi-nuclear MRS is most frequently employed using the phosphorus (^31^P) or carbon (^13^C) nuclei. Phosphorus is of physiological interest as high-energy phosphates play an important role in metabolic turnover and can be detected directly [84]. In particular, adenosine triphosphate (ATP) turnover and proton handling can be studied by ^31^P MRS of muscle [101,102], rate constants of changes in phospho-creatine (PCr) concentration during exercise [103] and, especially, recovery from exercise [101,104] can teach us about mitochondrial function and oxidative ATP re-synthesis [105,106]. As has been shown recently, a combination of different multi-nuclear MRS methods can further increase their value beyond the sum of their respective contributions by adding complementary information to a view on metabolism not accessible to each of the methods alone [85,107,108].

Magnetic resonance at ultra-high field strength has many advantages, particularly for MR spectroscopy [1,2,109]: firstly, the signal intensity, which is often the key factor for the feasibility of a spectroscopy study, increases with field strength and secondly, the chemical shift dispersion, *i.e.*, separation of the signal components which are attributed to the different metabolites to be quantified, obeys a linear relationship with field strength. This stronger separation makes the identification of resonances easier, more reliable or even possible at all, as signals of individual metabolites may not be adequately resolved at lower field strength. Additionally, the attraction of ultra-high field MRS is enhanced by the reduction of *T*_1_ relaxation times for certain metabolites (e.g., phospho-creatine) [110,111], allowing shorter repetition times and thus a further increase in signal-to-noise ratio (SNR) by averaging measurements, increasing time resolution or reducing total measurement time.

To fully exploit the advantages of increased magnetic field strength some technical challenges inherent to the MR physics in this regime need to be addressed:

- Higher demands on RF power, due to the higher resonance frequency, leads to increased specific absorption rate (SAR).
- Susceptibility effects increase with field strength, leading to broadening of peaks in spectra. Thus, MRS at higher field makes higher demands on shimming, (*i.e.*, the optimization of the local field homogeneity).
- The increased spectral dispersion at higher fields also requires correspondingly increased bandwidths of radio frequency pulses. This can be accomplished by shortening the pulse length while increasing the amplitude of the transmitted radio frequency’s magnetic field component. This in turn increases SAR and may lead to exceeding the maximum voltage which can be applied to the RF transmit coil.
- The nuclear resonant frequency increases linearly with static field strength, shortening the wavelength of applied RF pulses. This in turn gives rise to a highly inhomogeneous RF excitation.

## 2. Results and Discussion

Typical parameter settings for 3 T and 7 T protocols, based on our current experience on Siemens MR-systems, are described in the Experimental Section and exemplary results are shown below for various human organs, including brain, joints and cartilage.

### 2.1. High-Resolution Structural MRI

**Figure 1.**
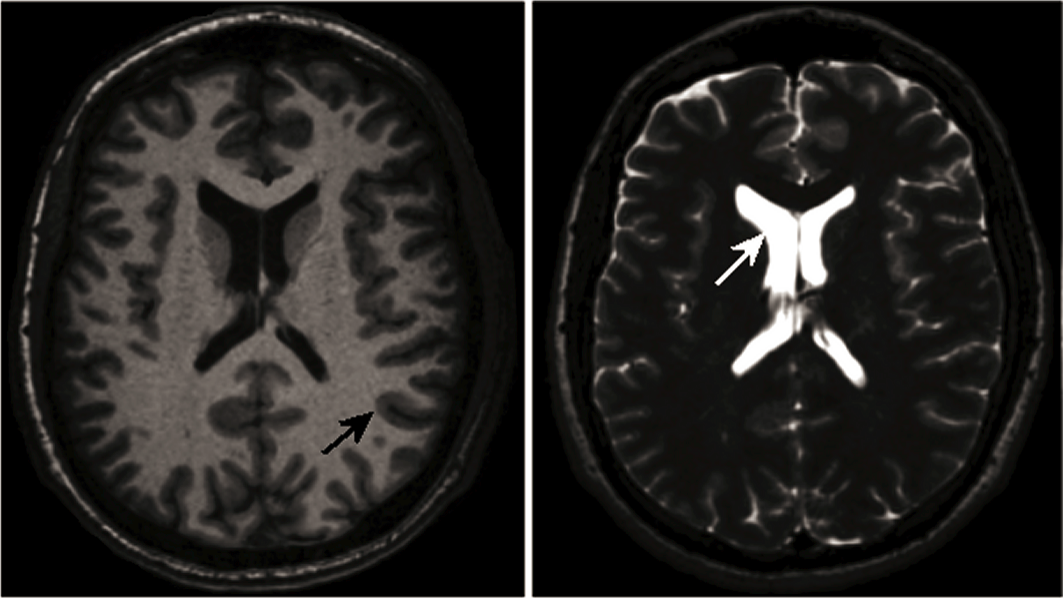
Comparison of T_1_ and T_2_ contrast at 3 T (12-channel head coil): T_1_-weighted, transverse slice through the brain (left). As can be seen T_1_-weighted images provide an excellent contrast between gray and white matter (black arrow) as well as a good definition of the ventricles. On the right side the same slice with T_2_-weighted contrast is shown; note the lower gray and white matter contrast but the excellent definition of the ventricles (white arrow).

Examples for high-resolution structural MRI of the brain are given in Figure 1. These images where acquired on a healthy volunteer and demonstrate T_1_ and T_2_ weighted contrast. Note the strong contrast between gray and white matter in the T_1_ weighted image as well as the excellent contrast between CSF and brain tissue in the T_2_ weighted image.

**Figure 2.**
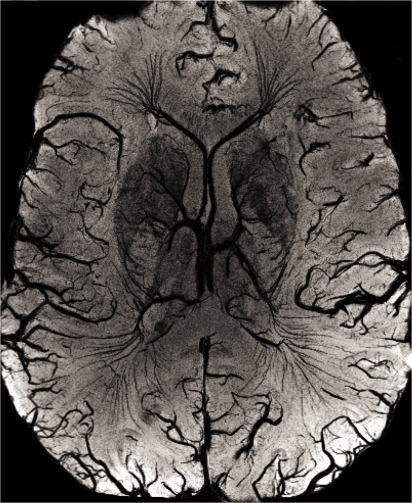
Susceptibility-Weighted Imaging at 7 T (8-channel head coil); this image represents a minimum intensity projection over a 6 mm slab. Note that both, veins and iron containing structures like the basal ganglia appear hypo intense. This image was acquired using an echo time of 15 ms and a resolution of 0.3 × 0.3 × 1.2 mm.

An example of susceptibility-weighted contrast in the human brain at 7 T is given in Figure 2. Note the excellent definition of veins, due to deoxygenated blood, as well as the good delineation of the basal ganglia, due to higher iron content.

**Figure 3.**
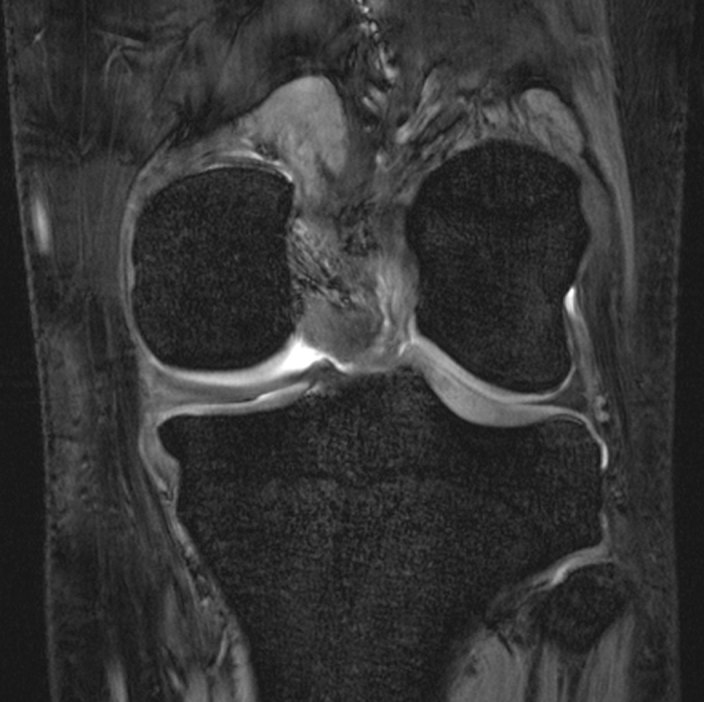
7 Tesla gradient-echo image of the knee joint in the coronal plane. The cartilage layers and the menisci are shown in high resolution and exquisite detail and will help to detect even subtle pathologies of these structures. Additionally, the trabecular structure of the bones is shown in ultra-high resolution, which may promote osteoporosis research.

**Figure 4.**
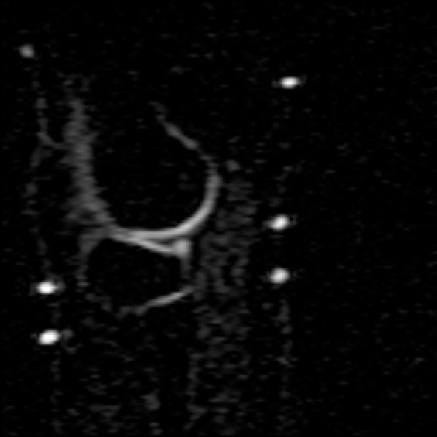
The 7 Tesla sodium image in the sagittal plane shows cartilage with high sodium content in the lateral femoral tibial joint cavity of the knee joint. Since sodium content correlates with the proteoglycan content of cartilage, which is related to the biomechanical properties of cartilage, a sodium image of cartilage provides biomechanical information. Note that even the thin cartilage layers of the proximal tibio-fibular joint are shown with sodium imaging.

Examples for high-resolution structural MRI of the knee and cartilage are given in Figure 3. A dedicated knee-coil was used to visualize details in cartilage and menisci. In addition to structural proton imaging, we have recently applied sodium imaging in patients after matrix-associated autologous cartilage transplantation (MACT). The resolution achieved was sufficient to visualize even the thin cartilage layer adjacent to the proximal tibio-fibular joint (Figure 4). In our patient group sodium imaging allowed to differentiate between different concentrations of sodium and hence GAG in different portions within the transplants which underscores its strength in the overall evaluation of cartilage transplants in contrast to the limited biopsy sample evaluation. We found a good correlation between sodium imaging and dGEMRIC in the quantification of the GAG concentration in patients after MACT [112].

### 2.2. Functional MRI

**Figure 5.**
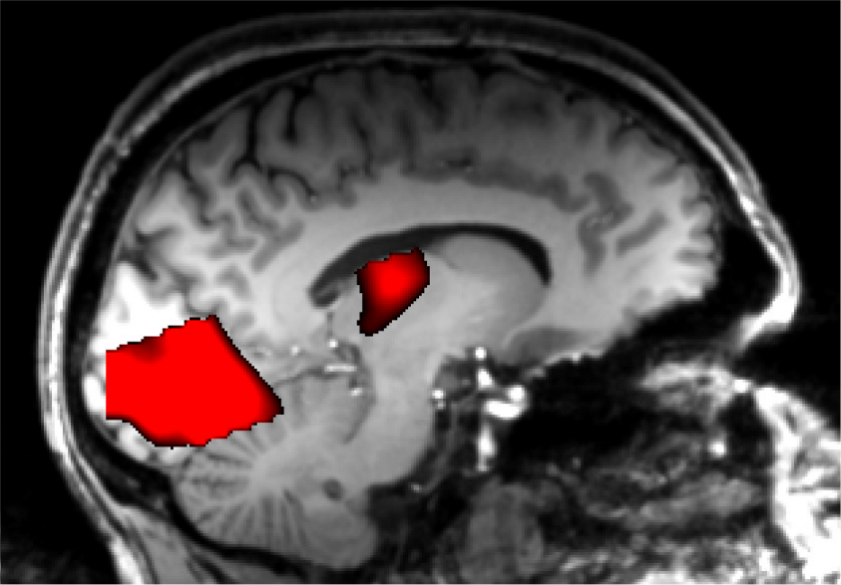
Brain regions that are activated by the different, ultra-short presentation durations using a *linear* regressor comprising the presentation durations of 1, 3, 5, 7, 9, and 11 ms. The sagittal map shows significant BOLD-signal increases projected onto the anatomical brain image of the subject (p < 0.05 FWE-corrected).

**Figure 6.**
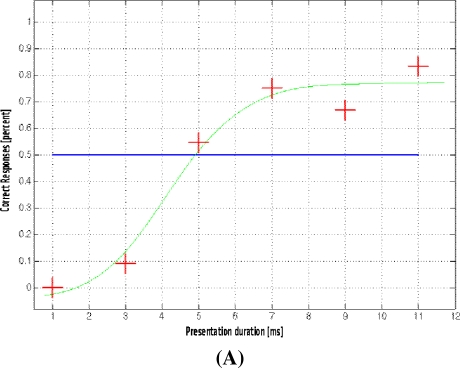

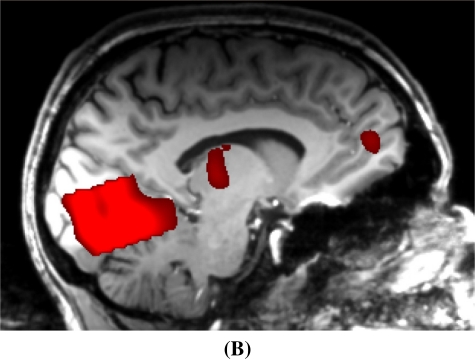
Activations associated with subjective perception. For this analysis a *sigmoidal* regressor corresponding to the probability with which the faces’ gender was detected (A) was used. The sagittal map (B) shows significant BOLD-signal increases projected onto the anatomical brain image of the particular subject (p < 0.05 FWE-corrected).

In this particular study healthy subjects were examined while performing a forced choice detection task. The subjects’ task was to identify black/white target faces, which were sequentially embedded within two pictures of equiluminiscent white noise. Targets were randomly chosen images from six equiluminiscent faces of men and women. Following each presentation, subjects were requested to rate if they were able to identify the faces’ gender, saw a face but could not rate the gender, or had not seen any presentation at all. Presentation durations were adjusted to three subliminal and three superliminal values according to the subjects’ individual visual sensory thresholds, and were presented 12 times each in randomized order. Individual thresholds were determined before fMRI scanning at 7 T. For each subject the percentage of correct detections per duration was calculated and finally a logistic curve fitted across all durations. Individual thresholds were defined as that duration which resulted in 50 percent correct recognitions. Exact timing of the presentations was provided by a custom build LC-shutter tachistoscope [113]. For single subject analyses statistical parametric maps were calculated using the general linear model with both regressors corresponding to the six presentation durations as well as sigmoidal regressors corresponding to the probability with which the faces’ gender was detected. This approach enabled on the one hand identification of those neuronal regions that were activated by the different presentation durations (Figure 5) and on the other hand identification of those brain areas that were involved in subjective perception (Figure 6). As expected, neuronal areas related to the different presentation durations were the thalamus and the primary visual cortex and areas that followed the perception curve were within the prefrontal and the cingulate cortex, additionally to the predominantly sensory areas.

Understanding the neuronal correlates of consciousness is one of the most challenging problems within the Cognitive Neurosciences. As mentioned above, probing stimuli around their sensory/perceptual thresholds is one way to identify brain structures that are essential for conscious experience. However, responses to stimuli presented for these short durations are weak and so is their BOLD response. In such applications the high sensitivity of a 7 T MR scanner is quite advantageous as can be seen from preliminary results presented here.

The brain areas identified with the two analysis approaches employed are in line with the so-called global neuronal workspace model [58]. Central to this theory is the hypothesis that two clearly distinguishable hierarchy levels can be described within the functional and anatomical architecture of the brain. The first level includes cortical and subcortical areas responsible for specific tasks within a certain sensory modality. According to the Dehaene model these areas are activated automatically and therefore work mostly unconscious. The primary visual cortex and the thalamus may be seen as part of this level since their activity linearly increased with presentation duration. The second level consists of prefrontal, cingulate, and parietal cortices and is characterised by many widespread connections to other areas within the brain. This high interconnectivity allows for fast and flexible exchange of information between the different sensory modules, a prerequisite for awareness. Additional activities within the prefrontal and cingulate cortex, as shown by the second analysis, confirms this view.

### 2.3. Multi-Modal Integration of MRI, fMRI, and Slow Cortical Potentials (SCP, DC-EEG)

In order to demonstrate the feasibility of simultaneous EEG and fMRI data acquisition a simple experiment using visual stimulation will be presented. As stimulus a slowly rotating checkerboard-propeller was presented in the center of the visual field for 8 s. Stimuli were repeated 40 times with a fixed 7 s black screen inter-stimulus interval in two runs, with and without parallel fMRI acquisition. EEG was recorded via 61 silver/silver chloride scalp electrodes using a NEURO PRAX® MR full-band EEG amplifier (NeuroConn Inc., Ilmenau, Germany). For offline eye movement artifact correction vertical and horizontal electrooculograms were recorded bipolarly from above and below the left eye and from the left and right outer canthi. The subject’s skin was slightly scratched at all recording sites in order to minimize skin potential artifacts and to ascertain homogeneous electrode impedance. The electrodes were filled with degassed electrode gel (Electro-Gel, ElectrodeCap International Inc., Eaton/OH, USA) to prevent ultra-slow baseline shifts [114]. Gradient and pulse artifacts were suppressed on-line using an adaptive template-matching algorithm. To this end up to 100 channel-specific templates were generated in a previous acquisition run [115]. Thereafter eye movement artefacts were eliminated using a linear regression approach with channel-specific correction coefficients [116]. Finally, single trial signals were low-pass filtered at 8 Hz and averaged. In order to identify the cortical structures involved, the resulting SCPs were subjected to current density analyses using sLoreta [61].

**Figure 7.**
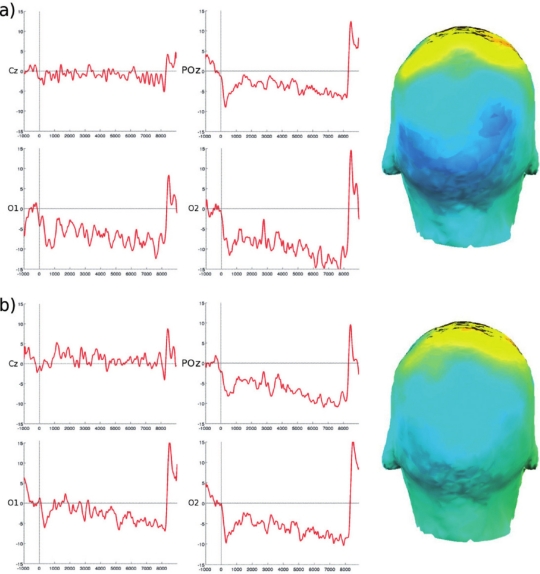
Averaged waveforms at representative scalp locations (left and middle row) and scalp potential topographies (right; dark blue color indicates stronger activation) averaged across the interval from 2,000–5,000 ms post stimulus of a single subject. (a) SCPs recorded inside the scanner without fMRI acquisition, (b) SCPs recorded while acquiring fMRI data.

**Figure 8.**
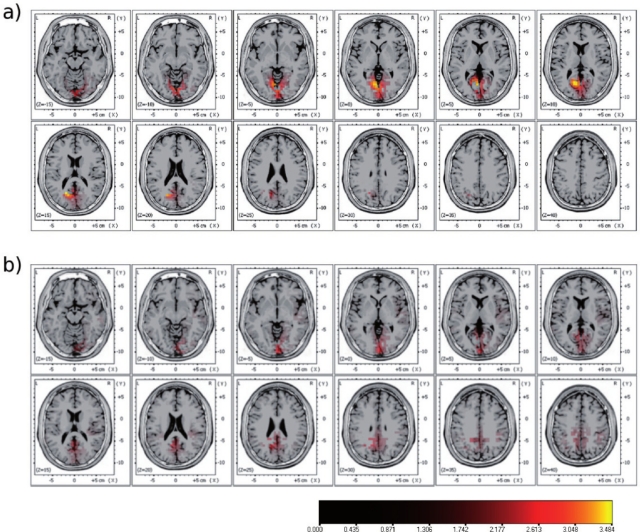
SCP-based source localization within the interval 2,000—5,000 ms post stimulus of a single subject. (a) SCPs recorded inside the scanner without fMRI acquisition, (b) SCPs recorded while acquiring fMRI data.

**Figure 9.**
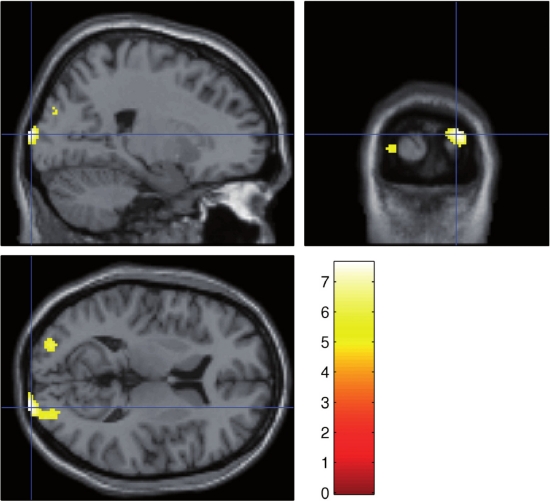
Single-subject fMRI results of the visual stimulation paradigm as used in Figures 7b and 8b (p < 0.001, uncorr.).

MR-images were acquired on a 3 Tesla TIM Trio system. For single subject analysis statistical parametric maps were calculated using the general linear model with regressors corresponding to the visual stimulation onsets. Activity in occipital areas was found in the SCP recordings of both acquisition runs (see Figure 7). A clear negative going SCP can easily be identified at occipital recording sites, which is slightly smaller during fMRI acquisition. As expected this waveform is restricted to sensory visual areas, *i.e*., to parietal and occipital recording sites, which can also be seen in the corresponding topographic potential pattern (color coded in blue). SCP based source localization (Figure 8) revealed strong activation in and around the primary visual cortex. The corresponding fMRI results showed activation primarily in the middle occipital gyrus (Figure 9).

The experiment presented resulted in SCP waveforms, topographies, and source localizations, comparable to data acquired outside the MR scanner. These results clearly demonstrate that simultaneous acquisition of DC-EEG and fMRI data, *i.e.*, data acquired by means of different sensor systems within one experiment covering different aspects of brain activity, is feasible at high magnetic fields.

### 2.4. Magnetic Resonance Spectroscopy

**Figure 10.**
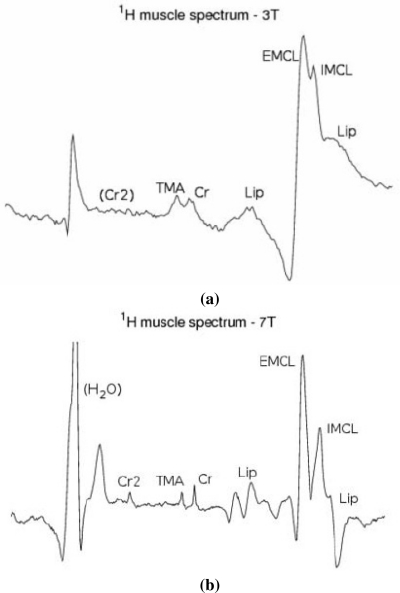
^1^H spectra of skeletal muscle acquired at 3 T (a) and 7 T (b), demonstrating improvement of spectral quality with increasing *B*_0_ field strength. Advantages of higher *B*_0_ field strength are improved separation of resonances and higher signal to noise ratio (b).

For an illustration of ultra-high field MRS applied to human skeletal muscle see Figure 10. Localized ^1^H spectra were acquired from human soleus muscle, at 3 and 7 Tesla, using a STEAM (“stimulated echo acquisition mode”) singel voxel localization scheme with an echo time *T_E_* = 20 ms and a voxel size of approx. 2 mL, in MR systems from the same manufacturer (Tim Trio and Magnetom 7 T, both by Siemens Medical Solutions, Erlangen, Germany). Parameters at 3 Tesla were *T_E_* = 20 ms, *T_M_* = 30 ms, *T_R_* = 4 s, VOI = 12 × 12 × 12 mm, 32 averages. At 7 Tesla, measurement parameters were *T_E_* = 20 ms, *T_M_* = 10 ms, *T_R_* = 1.5 s, VOI = 12 × 12 × 20 mm, 32 averages. Proton (^1^H) MR spectra of skeletal muscle allow quantification of e.g., total creatine, trimethylammonium compounds and lipids. Due to a bulk magnetic susceptibility effect, it is possible to distinguish between intra- and extracellular lipids, which becomes more pronounced at 7 Tesla. Several further advantages of ultra-high field strength, as compared to spectra acquired at 3 Tesla, become evident from the example given in Figure 10. As chemical shift dispersion increases linearly with *B*_0_, the separation of resonances improves (e.g., between TMA and creatine), provided that macroscopic field inhomogeneities can be compensated using high order shim routines. Signal-to-noise ratio is increased at higher field, improving reliability of quantification, for example the signal attributed to the CH_2_ group of creatine (Cr2) is clearly visible at 7 T.

**Figure 11.**
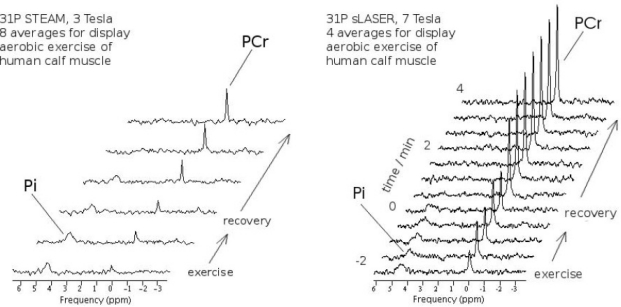
Dynamic localized ^31^P MRS of skeletal muscle acquired at 3 Tesla (left) and 7 Tesla (right). Signal gain at 7 T (in connection with an improved acquisition method, ^31^P semi-LASER) allows higher time resolution and yet surpasses spectral quality for quantification of creatine phosphate (PCr) and inorganic phosphate [87] time courses during and after aerobic exercise of human muscle, compared to a similar measurement at 3 T. Spectra were scaled to equal noise level.

Another example of ultra-high field MRS is shown in Figure 11. Dynamic localised ^31^P spectra of skeletal muscle were acquired on a 3 T whole body scanner (Bruker Biospin, Ettlingen, Germany) and a 7 T system (Siemens Medical Solutions, Erlangen, Germany). Signal gain at 7 Tesla—further improved here by using an advanced acquisition method which was recently implemented on the Siemens platform (^31^P semi-LASER, “localization by adiabatic selective refocusing” [104,117])—was utilized to achieve higher temporal resolution by avoiding averaging of consecutively acquired spectra. In exercising muscle, phosphocreatine is depleted under concomitant accumulation of inorganic phosphate. During recovery, PCr is resynthetised via oxidative phosphorylation, with a time constant typically on the order of minutes. These processes can be followed by dynamic ^31^P MRS. Measurements at 3 Tesla were performed with STEAM (*T_E_* = 7.5 ms, *T_M_* = 30 ms, *T_R_* = 7.6 s, 1024 complex data points per acquisition). At 7 Tesla, semi-LASER was used (*T_E_* = 53 ms, *T_R_* = 8 s, 2048 complex data points). The quality of 7 T data shown for each time point surpasses the quality of measurements at 3 T, so that no averaging was necessary at all for quantifying PCr in the 7 T spectra using a time domain fit routine for MR spectra. Reliable quantification of phosphocreatine (PCr) and inorganic phosphate [87] time courses during and after aerobic exercise of human muscle [84], from localized ^31^P spectra is thus possible with higher temporal resolution at 7 T. This allows determination of PCr resynthesis kinetics, a measure for oxidative capacity of skeletal muscle, with improved specificity.

## 3. Experimental Section

**Figure 12.**
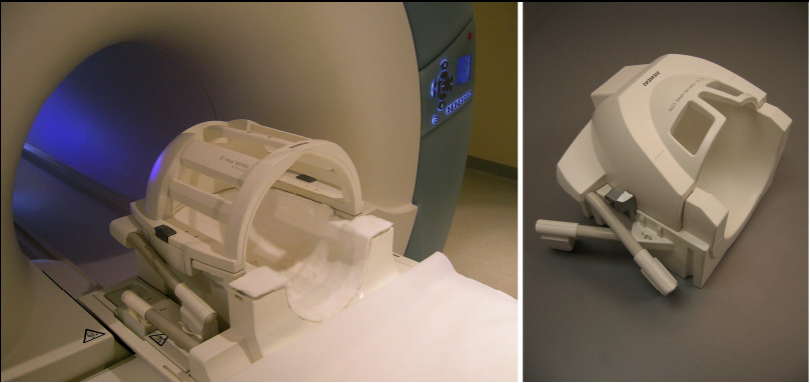
High field whole body MR scanner Siemens TIM Trio (3 Tesla, left) with a 12-channel receive-only head coil (Siemens). A cylindrically polarized (CP) whole body RF coil for transmitting 123 MHz (^1^H at 3 T) radio frequency signals and *B*_0_ gradient coils are installed in the scanner bore. Various Rx or local Tx/Rx coils can be used, e.g., a 32-channel receive head coil also by Siemens (right).

**Figure 13.**
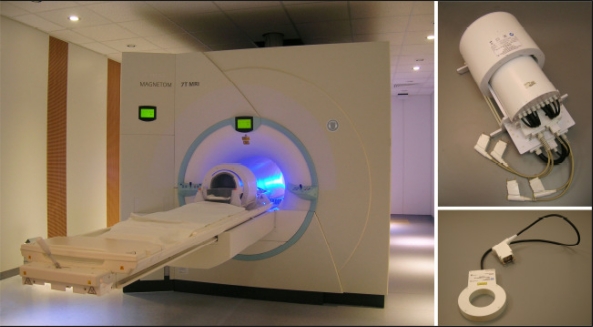
Ultra-high field whole body MR scanner Siemens Magnetom 7 T (left) with 24-channel (transmit-) receive coil (by Nova Medical) placed on the patient bed. *B*_0_ gradient-coils are installed in the scanner bore. The 24-channel Rx head coil (top right) is equipped with a transmit coil (outer cylinder), tuned to 300 MHz (Larmor frequency of ^1^H at 7 T). Single-loop transmit/receive surface coil by RAPID Biomedical (bottom right).

Here we briefly describe hardware and software requirements and parameters needed to run morphological, functional and metabolic MR-measurements and how to process the data. Basically, we need to modify the magnetic environment of the body and body molecules using a strong, static magnetic field and weak ‘gradient’ magnetic fields. The typical range of static magnetic fields in clinical systems is 0.2 T to 3.0 T (Figure 12); for human studies between 4 T and 9.4 T only research systems are available (Figure 13). Gradient coils typically produce relatively weak linear, orthogonal magnetic fields gradients of up to about 40 mT/m for the whole body or up to 100 mT/m for the head. Furthermore, adapted physical sensors (RF coils; see figures 12 and 13) are required to actually excite the nuclear spins in biomolecules (using a circular polarized magnetic field) in the region-of-interest (e.g., the brain), and detect weak RF signals from the tissue. Depending on the particular hardware used (*i.e.*, magnetic field strength, nucleus/resonance frequency chosen, gradient strength, duration and timing of RF-excitation pulses, *etc*.) a computer will create an image or spectrum of the signals detected [118]. Typically, these raw data will be processed to further reduce artifacts originating from physiological motion (e.g., from breathing, heart beat, gross body motion, susceptibility, flow, *etc*.), reconstructed again and either visually diagnosed or further processed using dedicated software and statistics. Contrast in the images obtained and used for diagnostic purpose is either native (*i.e.*, optimized via MR parameter settings) or enhanced via specific contrast agents (*i.e.*, by locally manipulating the magnetic environment using chemical sensors, e.g., chelates of gadolinium or iron particles).

The wavelength of applied RF pulses is reduced at higher field strength and in biological tissue (compared to air or vacuum) by its high relative permittivity, producing a proton resonant wavelength of approximately 12 cm inside the human body at 7 T. This is smaller than the dimensions of a typical organ (human head, thigh or abdomen), giving rise to strong interference within the sample, producing a highly inhomogeneous RF excitation [119–121]. The responsible effects are wavelength interference [121] and attenuation of RF amplitude by tissue conductivity [122]. Two main approaches to reducing this inhomogeneity have been proposed: RF shimming [123] and parallel transmission (pTx) [124–127], both of which rely on specific RF coil design and hardware extensions to the scanner. A full parallel Tx system enables transmission of completely independent RF pulse envelopes on all channels, while only amplitude and phase control is required for RF shimming. The RF excitation coils used for pTx consist of multiple independent transmission elements (typically 8 or 16), which have unique spatial profiles. These profiles may be modulated and superimposed to tailor the magnitude and phase of the excited magnetization [128] in the desired volume. Specific absorption rate varies by orders of magnitude, locally and on average, depending on the pattern of excitation. To ensure safety compliance, the complex behavior of power distribution needs to be validated in numerical models, accounting for target patterns and corresponding pTx pulses [124].

### 

#### 

##### Safety considerations:

As a diagnostic technique based on non-ionizing radiation, magnetic resonance imaging and spectroscopy can be considered a low risk procedure, as long as several safety-relevant parameters are monitored and kept within certain limits. Three main potential sources of hazards are connected with the use of magnetic resonance systems: (1) The radio frequency (RF) field, *B*_1_, required to tilt the magnetization vector from its equilibrium value and thereby induce a signal which can be measured, causes energy deposition in the tissue, (2) rapidly switched *B*_0_ field gradients, mainly applied for the purpose of encoding spatial information the MR signal, may lead to the induction of voltages in tissue, potentially causing nerve stimulation, and finally (3) the static magnetic field *B*_0_ poses a source of risks, predominantly due to the strong attraction forces on ferromagnetic objects. The potentially hazardous effects connected with *in vivo* magnetic resonance are subject to legal limits and recommendations issued by national and international organizations. Particularly, the United States Food and Drug Administration (FDA), the International Commission on non-ionizing Radiation Protection (ICNIRP), and the International Electrotechnical Commission (IEC) publish standards and guidelines defining limits of applicable RF-energy, the so-called specific absorption rate (SAR), magnetic gradient amplitudes and switching times and *B*_0_ field strength. Regulations limit the specific power absorption and/or resulting temperature increase [129] and define distinct thresholds for acceptable deposition, depending on the parts of the human body investigated. Limits distinguish between total average and average per gram of tissue in the head, abdomen and extremities, as well as averages on various time scales (typically over 5 or 6 min or short term averages of several seconds). It can be shown that the deposited power is proportional to the square of the static magnetic field *B*_0_^2^ and to the square of the irradiating RF field *B*_1_^2^ [130,131]. The calculation of the deposited energy is complex [122] due to the geometrical distribution pattern of *B*_1_ in biological tissue, particularly at high field strengths, where the wavelength of the RF wave in tissue is on the order of (or smaller than) the dimensions of the organs.

#### Technical Details on MRI Protocols:

##### Structural brain imaging:

*MP-RAGE parameters at 3 T*: TR/TI/TE = 2,300/900/3.59 ms, image-matrix = 320 × 320, 208 sagittal slices, GRAPPA factor = 2, resulting in an isotropic resolution of 0.8 mm, and scan time: 12.18 minutes.,

*MP-RAGE parameters at 7 T*: TR/TI/TE = 4,660/1700/4 ms, image-matrix = 320 × 320, 228 sagittal slices, GRAPPA factor = 2, resulting in an isotropic resolution of 0.65 mm, and scan time: 12.50 minutes.

*TSE parameters at 3 T*: 2D-Turbo Spin Echo (TSE), GRAPPA factor = 2, TR/TE = 13,070/92 ms, MA = 256 × 256, slices = 128, resulting in a resolution of 1 × 1 × 1.2 mm, and scan time of 5.15 minutes.

*Advanced cartilage imaging at 7 T*: PD_FSE; TR = 2,400 ms, TE = 24 ms, 36 slices, FOV = 120 cm^2^, Matrix = 1024 × 1024, slice thickness: 2 mm, scan time: 6min37sec.

*SWI parameters at 3 T*: A three-dimensional, fully first-order flow-compensated GE sequence with a TE of 29 ms; TR = 36 ms; image-matrix = 320 × 320 pixel; slices = 104; parallel imaging (GRAPPA) factor = 2; resolution = 0.64 × 0.64 × 1.3 mm.

*SWI parameters at 7 T*: SWI sequence; TE = 15 ms; repetition time = 28 ms; image-matrix = 704 × 528 pixel; slices = 96; parallel imaging (GRAPPA) factor = 2; scan time = 9.48 min; resolution = 0.3 × 0.3 × 1.2 mm^3^.

##### Functional brain imaging:

*FMRI-EPI parameters at 3 T*: single-shot gradient-recalled echo-planar imaging; 12 axial slices (4 mm thickness, 3 mm gap) aligned to the connection line between anterior and posterior commissure; matrix size = 64 × 96; FOV = 210 × 210 mm^2^, and TE/TR = 42/1,000 ms.

*FMRI-EPI parameters at 7 T*: single-shot gradient-recalled echo-planar imaging; 4 axial slices (5 mm thickness), aligned to include the thalamus and the fusiform gyrus, matrix size = 128 × 128; FOV = 240 × 240 mm^2^, and TE/TR = 26/200 ms.

*^23^Na-MRI parameters at 7 T*: Modified 3D-GRE sequence: TR/TE = 10.0/3.77 ms; FOV = 199 × 199 mm^2^, 48 slices; MA = 64 × 128; resulting in a resolution of 3.11 × 1.55 × 3.0 mm^3^, and scan time of 30:45 min.

## 4. Conclusions

The major advantage of modern sensor technology, including higher magnetic fields, multi-array transmit and receive coils, measurement techniques and contrast agents, is the essential shift from morphological to biochemical and metabolic imaging techniques, which typically suffer from low sensitivity at standard field strength (1.5 T). The high signal-to-noise ratio provided by the higher field systems, combined with novel sensors provides biochemical and metabolic imaging in reasonable scan times, which promotes its widespread clinical application. As a rule of thumb one could argue that all measurement techniques limited by SNR, but not SAR, at 1.5 T or 3 T should perform better at higher magnetic fields. This includes high-resolution 3D-MRI of small structures, high-resolution fMRI, and localized 31P-MRS techniques. On the other hand, there is no need to transfer methods which are already limited by SAR and which are clinically useful at 1.5 T or 3 T to higher fields. This is not to say that no particular contrast mechanisms and novel approaches may be developed especially at very high fields and may find also clinical use. For MR methods in which the underlying contrast mechanism does not depend on magnetic field strength, such as water diffusion or gadolinium based contrast agents, no substantial gain can be expected except from a general increase in SNR. Finally it will be the best possible combination of organ size and location, tissue of interest structure and pathology, Tx/Rx coil sensitivity and homogeneity, contrast mechanism, image protocol and data processing strategy employed which will provide the best data quality available in a given time. In addition, artifact reduction during preprocessing and appropriate modeling during post-processing may further enhance diagnostic quality. This development would, for example, allow a shift towards a diagnosis of diseases in their earliest stages such as osteoarthritis and degenerative disc disease, for instance, before severe morphological changes in proton-MRI occur. Thus, it may be stated that the basic imaging requirements are already available for the evaluation and follow-up of new disease modifying drugs leading to a more personalized medicine. Recent advances in sensor technology, *i.e.*, magnets, improved gradient performance, and multi coil RF technology may also enable one to achieve ultra short echo times (TE ≤ 0.2 ms) that can significantly improve resolution and SNR in sodium imaging. These advances may potentially make clinical sodium MRI feasible on 3 T scanners. Further, the recent increase in number of 7 T whole-body MRI scanners in clinical research centres could have a significant impact on sodium MRI and its potential for clinical use. Since SNR scales as B_0_^7/4^ [2,132,133] there is a lack of B_1_ penetration and B_0_ inhomogeneity, issues that pose serious problems with proton imaging, and sodium MRI could be particularly advantageous at higher fields. Thus, ^23^Na MRI at 7 T may become clinically useful and has the potential to become a new standard in biochemical imaging of articular cartilage with its direct correlation to glycosaminoglycan concentration, the component of the ultra-structure of cartilage, which is lost in the earliest stages of OA and plays the most important role in the biomechanical properties of cartilage. This would carry MR-based diagnosis from current ^1^H-MRI to techniques measuring parameters more closely related to tissue metabolism.

In summary, high-resolution ^1^H-MRI, SWI, fMRI, ^23^Na-MRI and ^31^P-MRS show great potential for further methodological improvements and both, research and clinical applications. A serious constraint, however, is posed by financial limits and capacity, including specialized training of scientists and physicians, which have to be addressed in order to not only develop potentially useful diagnostic sensors and techniques but also to make these suitable for as many people as possible, tailored to individual patient’s needs.
